# Body Mass Index is a Poor Predictor of Bedside Appendix Ultrasound Success or Accuracy

**DOI:** 10.5811/westjem.2016.5.29681

**Published:** 2016-06-29

**Authors:** Samuel H.F. Lam, Christopher Kerwin, P. John Konicki, Diana Goodwine, Michael J. Lambert

**Affiliations:** Advocate Christ Medical Center, Department of Emergency Medicine, Oak Lawn, Illinois

## Abstract

**Introduction:**

The objective of this study was to determine whether there is a relationship between body mass index (BMI) and success or accuracy rate of beside ultrasound (BUS) for the diagnosis of appendicitis.

**Methods:**

Patients four years of age and older presenting to the emergency department with suspected appendicitis were eligible. Enrollment was by convenience sampling. After informed consent, BUS was performed by trained emergency physicians who had undergone a minimum of one-hour didactic training on the use of BUS to diagnose appendicitis. We ascertained subject outcomes by a combination of medical record review and telephone follow up. Calculated BMI for adults and children were divided into four categories (underweight, normal, overweight, obese) according to Centers for Disease Control and Prevention classifications.

**Results:**

A total of 125 subjects consented for the study, and 116 of them had adequate image data for final analysis. Seventy (60%) of the subjects were children. Prevalence of appendicitis was 39%. Fifty-two (45%) of the BUS studies were diagnostic (successful). Overall accuracy rate was 75%. Analysis by chi-square test or Mann-Whitney U test did not find any significant correlation between BMI category and BUS success. Similarly, there was no significant correlation between BMI category and BUS accuracy. The same conclusion was reached when children and adults were analyzed separately, or when subjects were dichotomized into underweight/normal and overweight/obese categories.

**Conclusion:**

BMI category alone is a poor predictor of appendix BUS success or accuracy.

## BACKGROUND

In recent years studies have been published on the use of beside ultrasound (BUS) to diagnose appendicitis in the emergency department (ED).^1^–^4^ Its popularity is likely due to the improving ultrasound skills of emergency physicians, as well as the obvious BUS advantages of no ionizing radiation emission, and ease of performance and interpretation at the bedside. Use of ultrasound in suspected appendicitis is also supported by American College of Radiology recommendations, especially in the pediatric population.^5^

Body habitus can be a limiting factor in appendix ultrasound. Several studies have reported decreased ultrasound success rate and accuracy with increasing body mass index (BMI).^6^–^11^ Nevertheless, such findings are by no means universal.^12^–^15^ Furthermore, none of the studies was conducted with BUS performed in the ED setting.

The purpose of the current study was to determine whether there is a relationship between BMI and success or accuracy of BUS for the diagnosis of appendicitis.

## METHODS

This was a single-site, prospective study on patients treated at the Advocate Christ Medical Center Emergency Department for suspected appendicitis. It was approved by our institutional review board. The hospital is a community tertiary referral center with approximately 100,000 ED visits per year. The ED is staffed entirely by board-certified emergency physicians, and sponsors a three-year emergency medicine residency training program. On-site staff radiologists provide interpretation of radiologic studies at all hours.

Patients four years of age and older presenting to the ED with abdominal pain concerning for appendicitis (as determined by the ED attending physician after history and physical examination) were eligible for enrollment. Exclusion criteria included previous appendectomy, pregnancy, unstable vital signs, frank peritonitis, neurological deficits interfering with the ability to localize abdominal pain, wards of the state, and subject/guardian refusal of consent. Enrollment was by convenience sampling, depending on whether a study investigator was available. Investigators were emergency physicians who had undergone a minimum of one-hour didactic training given by the senior investigator (ML) on the use of ultrasound to diagnose appendicitis. Study investigators were allowed to simultaneously function as treating emergency physicians, and were not blinded to the presentation and clinical history of the subjects.

After informed consent, a focused clinical history and physical examination was obtained from each study subject, followed by an abdominal BUS performed with a Zonare Z. One (Mountain View, CA) or Sonsite M-Turbo (Bothell, WA) machine, using graded compression technique. Investigators concluded their BUS when, in their judgment, the best possible images in the subjects were obtained. All BUS studies were completed prior to any radiology department studies or surgical consultations. Patients were treated according to the judgment of the ED attending physicians or consultants.

Subject data collected included age, sex, height, weight, BMI, components of history and physical examination, and laboratory test results. Sonographic findings were recorded on the data collection form. Investigators’ overall impressions of the BUS, based on real-time sonographic findings at the bedside, were documented in the patients’ medical records.

Diagnostic test and imaging results, pathological reports, intra-operative findings, and subject hospital course, if available, were obtained by review of the medical record. A research nurse made follow-up telephone calls at 24 hours and 30 days to subjects who were discharged from the ED or who did not receive operative intervention. Three separate attempts to establish contact were made before subjects were deemed lost to follow up. Final patient outcome was adjudicated by one of the investigators (SL) based on the information obtained by the above-mentioned means.

All study information was recorded on patient data sheets, and then entered onto an Excel (2007, Microsoft Corp., Redmond, WA) spreadsheet for analysis. We divided calculated BMI for adults and children (18 years of age and younger) into four categories (underweight, normal, overweight, obese) according to United States Centers for Disease Control and Prevention (CDC) classifications. We defined adults as those over 18 years, instead of the CDC criterion of over 21 years, to conform to the standard in the prevailing appendix ultrasound literature. We analyzed data by SPSS (version 20.0, IBM Corp., Armonk, NY). BUS studies were considered successful when the operator was able to make the diagnosis of “appendicitis” or “no appendicitis” as recorded in the data entry form or the medical record. We calculated the accuracy of ED BUS studies using the outcomes above as the gold standard. Correlation between BMI and BUS success and accuracy were analyzed using chi-square test and Mann-Whitney U test.

## RESULTS

This study examines the relationship between BMI and BUS success and accuracy. A total of 125 subjects were consented, and 116 had adequate image data for final analysis. (Images on nine subjects failed to transfer to database after recording.) Mean age of the subjects was 20.2 years, and 51% were male. Sixty percent were 18 years of age or younger. Table 1 shows the distribution of subject BMI according to CDC classifications. Prevalence of appendicitis was 39%.

**Figure 1:**
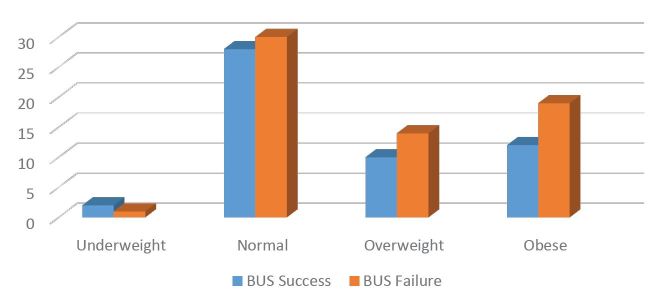
Bedside ultrasound (BUS) success rate categorized by body mass index.

Fifty-two (45%) of the 116 BUS studies were diagnostic (successful). Figure 1 and Table 2 illustrate the BUS success rate according to subject BMI categories.

**Figure 2:**
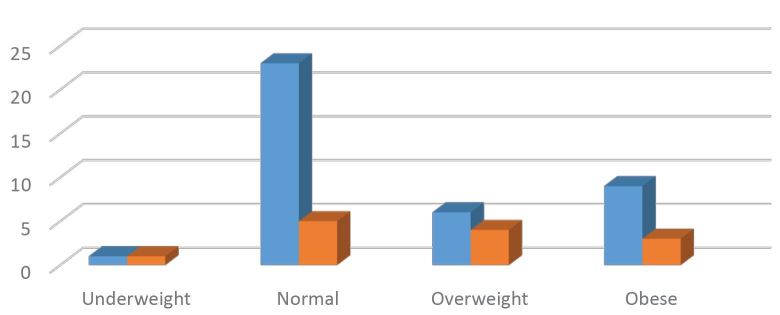
Beside ultrasound (BUS) accuracy rate categorized by body mass index

Among the diagnostic BUS studies, there were 33 true positive, 13 false positive, 6 true negative, and no false negative BUS studies. This corresponds to an overall accuracy of 75%. Figure 2 and Table 3 describe BUS accuracy categorized by BMI.

**Figure 3:**
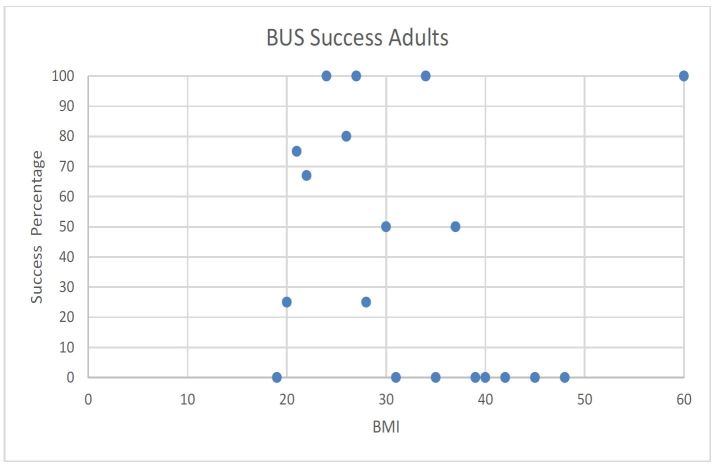
Beside ultrasound (BUS) success rate versus body mass Index (BMI) in adult patients.

**Figure 4:**
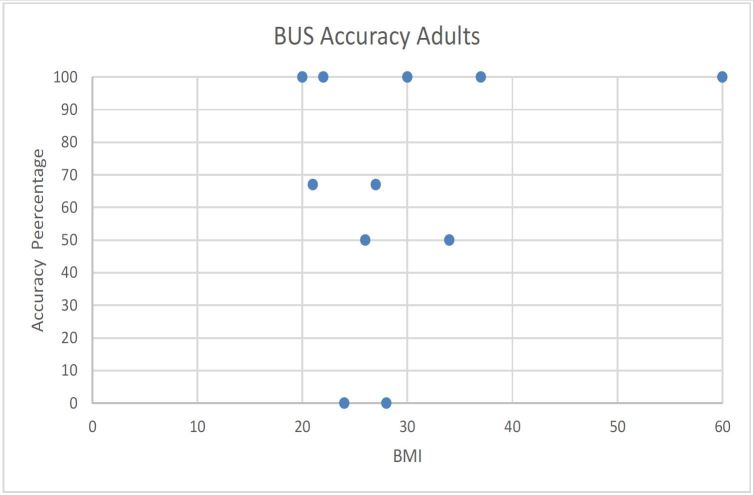
Beside ultrasound (BUS) accuracy rate versus body mass index (BMI) in adult patients.

**Figure 5:**
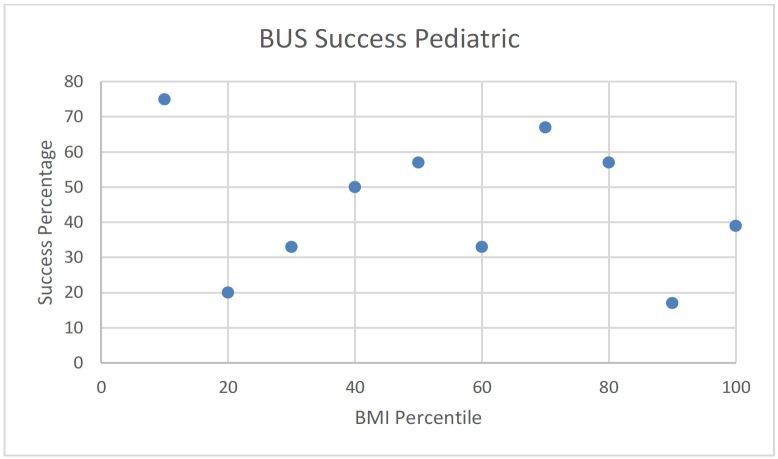
Beside ultrasound (BUS) success rate versus body mass index (BMI) percentile in pediatric patients.

**Figure 6:**
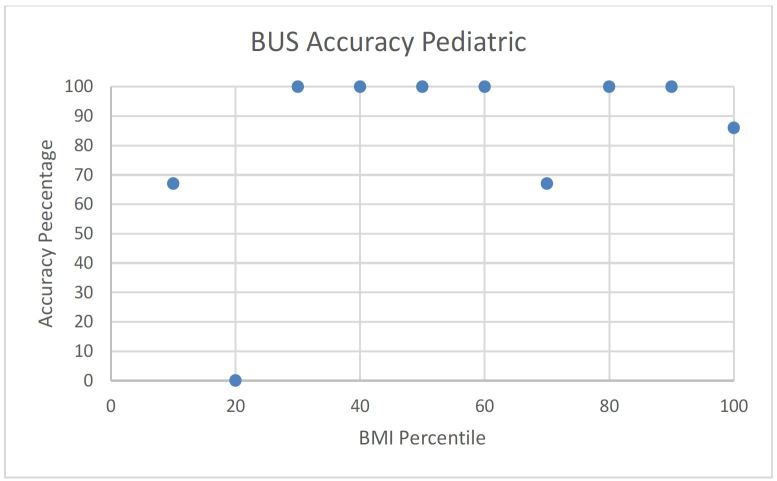
Beside ultrasound (BUS) accuracy rate versus body mass index (BMI) percentile in pediatric patients.

No obvious trend was observed when BUS success and accuracy was plotted against individual BMI/BMI percentile in adult and pediatric patients (Figures 3, 4, 5, and 6).

Statistical analysis by chi-square test or Mann-Whitney U did not find any significant correlation between BMI category and BUS success rate. Similarly, there was no significant correlation between BMI category and BUS accuracy. We reached the same conclusions when adults and pediatric populations were analyzed separately, or when subjects were dichotomized into underweight/normal and overweight/obese categories.

We also examined the outcome of the 64 subjects whose BUS was non-diagnostic. Twenty-eight of them underwent radiology department-performed ultrasound, with only nine studies interpreted as diagnostic. The overall accuracy of these nine studies (4 positives, 5 negatives) was 67% (2 false positives, 1 false negative). Forty-two of the subjects had abdominal and pelvis computed tomography performed, with an overall accuracy of 98% (1 false positive, no false negative).

## DISCUSSION

As far as the authors are aware, ours is the first study examining the relationship between BMI and accuracy and success rate of bedside appendix ultrasound performed in the ED setting.

Multiple studies have investigated the relationship between BMI and accuracy and success rate of radiology department-performed appendix ultrasound, and the conclusions have been inconsistent. Josephson et al. found that sensitivity (but not specificity or accuracy) of appendix ultrasound was significantly lower in patients with BMI≥25 compared with those<25.^6^ Their findings were echoed in a study by Blebea et al.^7^ On the contrary, Keyzer et al. found BMI had no effect on the accuracy or success rate of appendix ultrasound, regardless of the expertise of the performing radiologist.^12^ A recent study by de Oliveira Peixoto came to the same conclusion.^13^

Similarly, the topic has been researched in pediatric patients with mixed findings. Two studies found that children with BMI≥85^th^ percentile have lower appendix ultrasound accuracy,^8^,^9^ and two other studies found that obese children have lower appendix identification rate on ultrasound.^10^,^11^ Other studies have failed to find any relationship between BMI of children and accuracy^14^,^15^ or success^14^ of appendix ultrasound. Nevertheless, Abo et al. did observe a trend of decreasing ultrasound sensitivity with increasing BMI in their study of 176 children with suspected appendicitis.^14^

While it makes intuitive sense that increasing BMI might lead to decreasing appendix ultrasound accuracy and success due to generally poor penetration of the high frequency (5–15MHz) transducer commonly used for the application, it is likely not the sole determining factor. Operator experience, duration of symptoms (hence the degree of inflammatory changes present), ultrasound machine make and model, location of the appendix, and patient cooperation can all affect the outcome of such examination. Although no statistical significant relationship was found, we observed a trend that as BMI increased, appendix ultrasound success and accuracy declined to the degree of approximately10–20%. This magnitude of difference parallels those found in previously cited studies, whether statistical significance was found or not.^8^,^9^,^14^,^15^

BUS has been found to be moderately sensitive and specific in making the diagnosis of appendicitis.^1^–^4^ Given the relatively small impact BMI has on its diagnostic accuracy and success rate, and the obvious advantages of no ionizing radiation and potential facilitated clinical decision-making, we believe that BUS should be attempted in all ED patients presenting with suspected appendicitis, regardless of BMI, by clinicians who are trained in the application.

## LIMITATIONS

A major limitation of the study was convenience sampling of the subjects, leading to possible selection bias. Nevertheless, nearly half of our included subjects had BMI in the overweight or obese range, which would argue against patient selection according to body habitus by investigators. Investigators were unblinded to the history and clinical examination findings of the subjects. Awareness of these findings, however, is exactly what distinguishes BUS from ultrasound performed by non-clinicians. Hence, we do not consider this a weakness of our study. Our sample size was relatively small, limiting the power of our conclusions, and this was a single-center study. All investigators who performed BUS in our study were ED ultrasound fellows or faculty, with ultrasound experience exceeding that recommended by the American College of Emergency Physicians.^16^ Hence, our study findings may not be applicable to operators with different BUS skill levels. Study results might also be different in institutions using different point-of-care ultrasound machines than ours.

## CONCLUSION

We failed to demonstrate any significant relationship between body mass index and success or accuracy of bedside appendix ultrasound performed in the emergency department.

The authors wish to thank Kathleen Hesse, RN for her diligent followup of the study subjects, and for compilation of the study data spreadsheet. We also thank Christopher Blair, MS for providing statistical support and data analysis. We appreciate the help of Anna Kienicki-Sklar, MD and Cindy Chan, MD in recruitment of our study subjects.

## Figures and Tables

**Table 1:** Subject body mass index distributions according to Centers for Disease Control and Prevention classifications.

	Adult (%)	Children (≤age 18) (%)	
Underweight (<18.5)	0 (0)	3 (4)	Underweight (<5^th^ %tile)
Normal (18.5–24.9)	17 (37)	41 (59)	Normal (5–84.9^th^ %tile)
Overweight (25–29.9)	13 (28)	11 (16)	Overweight (85–94.5^th^ %tile)
Obese (≥30)	16 (35)	15 (21)	Obese (≥95^th^ %tile)

**Table 2:** Beside ultrasound (BUS) success rate categorized by body mass Index (BMI).

BMI category	BUS success (%)	BUS failure (%)
Underweight	2 (67)	1 (33)
Normal	28 (48)	30 (52)
Overweight	10 (42)	14 (58)
Obese	12 (39)	19 (61)

**Table 3:** Beside ultrasound (BUS) accuracy categorized by body mass index (BMI).

BMI category	BUS accurate (%)	BUS inaccurate (%)
Underweight	1 (50)	1 (50)
Normal	23 (82)	5 (18)
Overweight	6 (60)	4 (40)
Obese	9 (75)	3 (25)

## References

[1] Chen SC, Wang HP, Hsu HY (2000). Accuracy of ED sonography in the diagnosis of acute appendicitis. Am J Emerg Med.

[2] Fox JC, Hunt MJ, Zlidenny AM (2007). Retrospective analysis of emergency department ultrasound for acute appendicitis. Cal J Emerg Med.

[3] Fox JC, Solley M, Anderson CL (2008). Prospective evaluation of emergency physician performed bedside ultrasound to detect acute appendicitis. Eur J Emerg Med.

[4] Lam SHF, Grippo A, Kerwin C (2014). Bedside ultrasonography as an adjunct to routine evaluation of acute appendicitis in the emergency department. West J Emerg Med.

[5] Rosen MP, Ding A, Blake MA (2011). ACR Appropriateness Criteria® right lower quadrant pain—suspected appendicitis. J Am Coll Radiol.

[6] Josephson T, Styrud J, Eriksson S (2000). Ultrasonography in acute appendicitis. Body mass index as selection factor for US examination. Acta Radiol.

[7] Blebea JS, Meilstrup JW, Wise SW (2003). Appendiceal imaging: which test is the best?. Semin Ultrasound CT MR.

[8] Schuh S, Man C, Cheng A (2011). Predictors of non-diagnostic ultrasound scanning in children with suspected appendicitis. J Pediar.

[9] Taylor GA (2011). Ultrasound scan for suspected appendicitis in children: risk of diagnostic inaccuracy increases with BMI at or above 85th percentile and clinical probability of appendicitis of 50% or lower. Evid Based Med.

[10] Hormann M, Scharitzer M, Stadler (2003). Ultrasound of the appendix in children: is the child too obese?. Eur Radiol.

[11] Trout AT, Sanchez R, Ladino-Torres MF (2012). A critical evaluation of US for the diagnosis of pediatric acute appendicitis in a real-life setting: how can we improve the diagnostic value of sonography?. Pediatr Radiol.

[12] Keyzer C, Zalcman M, De Maertelaer V (2005). Comparison of US and unenhanced multi-detector row CT in patients suspected of having acute appendicitis. Radiology.

[13] Peixoto R deO, Nunes TA, Gomes CA (2011). Indices of diagnostic abdominal ultrasonography in acute appendicitis: influence of gender and physical constitution, time evolution of the disease and experience of radiologist. Rev Col Bras Cir.

[14] Abo A, Shannon M, Taylor G (2011). The influence of body mass index on the accuracy of ultrasound and computed tomography in diagnosing appendicitis in children. Pediatr Emerg Care.

[15] Yigiter M, Kantarci M, Yalcin O (2011). Does obesity limit the sonographic diagnosis of appendicitis in children?. J Clin Ultrasound.

[16] American College of Emergency Physicians (2009). Emergency ultrasound guidelines. Ann Emerg Med.

